# Effects of Narrative Exposure Therapy on Posttraumatic Stress Disorder, Depression, and Insomnia in Traumatized North Korean Refugee Youth

**DOI:** 10.1002/jts.22492

**Published:** 2020-03-26

**Authors:** Jinme K. Park, Jinah Park, Thomas Elbert, Seog Ju Kim

**Affiliations:** ^1^ Department of Psychology University of Konstanz Konstanz Germany; ^2^ Department of Counseling Kyonggy University Suwon Republic of Korea; ^3^ Department of Psychiatry, Samsung Medical Center Sungkyunkwan University School of Medicine Seoul Republic of Korea

## Abstract

Refugees affected by multiple traumatic stressors are at high risk for developing trauma‐related mental disorders, including posttraumatic stress disorder (PTSD), depression, and insomnia, which is sometimes overlooked. The present study examined the effectiveness of narrative exposure therapy (NET) on trauma‐related symptoms in a sample of North Korean refugee youth. We focused on sleep patterns in addition to changes in symptom severity for PTSD, depression, and internalizing and externalizing symptoms. North Korean refugee youth (*N* = 20) with PTSD were assigned to either an NET‐based treatment group or a control group, which consisted of treatment as usual (TAU). There were clinically significant reductions in PTSD, depression, and internalizing and externalizing symptoms for the NET group, Hedges’ *g* = 3.6, but not the TAU group. The change in diagnostic status for PTSD was more notable for participants in the NET group compared to the TAU group. Of note, NET also produced a significant improvement in insomnia symptoms and sleep quality, Hedges’ *g* = 2.1. The substantial recovery regarding overall posttraumatic symptoms in the NET group was observed 2 weeks after the end of treatment and remained stable at 6‐month follow‐up. The results of the present study suggest that NET may be a treatment option for traumatized North Korean refugee youth and may also be effective for the treatment of sleep problems that arise from traumatic experiences.

Displaced populations, such as refugees and asylum seekers, have often experienced multiple traumatic stressors (Augsburger, Dohrmann, Schauer, & Elbert, 2017; Ibrahim & Hassan, 2017). The more traumatic stressors an individual experiences, the higher the probability they will have trauma‐related mental disorders, including posttraumatic stress disorder (PTSD), depression, and insomnia (Chung et al., 2018; Hodges et al., 2013; Wang, Raffeld, Slopen, Hale, & Dunn, 2016; Wilker et al., 2015). Consequently, variable but generally high rates of PTSD and comorbid symptoms of depression and insomnia have been reported among refugees and war‐affected populations (Bogic, Njoku, & Priebe, 2015; Y. ‐J. Lee et al., 2016; Park, Elbert, Kim, & Park, 2019; Reavell & Fazil, 2017).

In recent reviews of psychological interventions for refugees and asylum seekers, trauma‐focused treatments have been found to be the most efficacious and promising interventions for treating PTSD in traumatized refugees (Nickerson, Bryant, Silove, & Steel, 2011; Nickerson et al., 2017). Nickerson et al. (2017) recommended further studies with refugees to investigate the efficacy of treatments in improving psychological symptoms beyond PTSD as well as broader outcomes of treatments. Limited evidence exists regarding whether cognitive behavioral treatment, such as prolonged exposure therapy (Brownlow et al., 2016), cognitive processing therapy (Gutner, Casement, Gilbert, & Resick, 2013), and cognitive therapy for PTSD (Woodward et al., 2017), improves sleep disturbances among traumatized individuals. However, the question of whether the high rates of insomnia among refugees with PTSD can be treated through trauma‐focused interventions has not yet been addressed.

Narrative exposure therapy (NET; Schauer, Neuner, & Elbert, 2011) can be effectively applied in the treatment of refugees, even in individuals with high levels of PTSD and insomnia. Narrative exposure therapy is a trauma‐focused intervention that was developed to address PTSD and mental distress resulting from multiple traumatic stressors. A large number of randomized controlled trials have suggested that NET decreases PTSD symptoms and other trauma‐related mental health symptoms, including those associated with depression and borderline personality disorder (BPD; Alghamdi, Hunt, & Thomas, 2015; Orang et al., 2018; Pabst et al., 2014; Robjant, Roberts, & Katona, 2017). However, to our knowledge, the only previous study to examine the effects of NET on various sleep parameters was conducted by Weinhold et al. (2017), who used a small sample of individuals with BPD and comorbid PTSD. Their results suggest that NET may reduce sleep onset latency and arousal. However, to date, no studies of which we are aware have examined the effect of NET on changes in sleep patterns among traumatized refugees.

North Korean refugee youth affected by organized violence and poverty in their home country or during flight have reported high rates of multiple trauma exposures and high levels of subsequent trauma‐related symptoms, including PTSD, depression, insomnia, and behavioral problems (Kim & Shin, 2015; Kim, 2016; Park et al., 2019). However, little is known about effective and useful evidence‐based interventions for treating traumatized North Korean refugee populations (Lee, Lee, & Park, 2017). There is a lack of published research that has examined the effects of trauma‐focused interventions on PTSD in North Korean refugee youth, specifically research that compares such therapies with other intervention approaches. The present study aimed to test the extent to which trauma‐related symptoms can be addressed by trauma‐focused treatment in a sample of North Korean refugee youth. We compared NET to treatment as usual (TAU), focusing on insomnia and sleep quality in addition to changes in symptoms of PTSD and depression. We also examined changes in internalizing and externalizing symptoms. We hypothesized that NET would result in a significantly larger reduction in clinical symptoms and sleep problems compared to TAU.

## Method

### Participants and Procedure

Participants were 20 North Korean refugee youth with a mean age of 19 years (range: 16–24 years). Individuals were assigned to either the NET (*n* = 9) or TAU group (*n* = 11). Assignment to groups followed convenience in the organizational process due to its environmental constraints and independent of individual characteristics. The two groups did not differ regarding sociodemographic characteristics or the number of traumatic events experienced. Regarding mental health symptom indices, significantly higher values for depression symptom severity were found among participants in the TAU group (see Table 1). One participant had received outpatient pharmacological treatment for probable attention deficit and hyperactivity disorder at the start of the study, and this individual's medication was kept constant during the study.

**Table 1:** Sociodemographic Characteristics and Mental Health Symptom Indices

	NET		TAU		
	(*n* = 9)		(*n* = 11)		
Variable	*M*	*SD*	*M*	*SD*	*p*
Age (years)	18.89	1.05	18.73	2.72	.869
No. of traumatic event types	5.44	1.59	5.73	1.62	.700
Mental health symptoms					
PTSD	48.0	6.44	49.45	10.13	.714
Insomnia	16.67	4.72	18.09	4.74	.512
Sleep quality	12.11	2.89	13.36	3.11	.367
Depression	13.0	6.0	17.64	3.5	.045
Internalizing and externalizing symptoms	16.78	5.24	19.18	3.46	.234

*Note*. NET group: *n* = 6 girls and women, *n* = 3 boys and men; TAU group: *n* = 9 girls and women, *n* = 3 boys and men. NET = narrative exposure therapy; TAU = treatment as usual; PTSD = posttraumatic stress disorder.

The study was approved by the Ethical Review Board of the University of Konstanz and the Samsung Medical Center Institutional Review Board, and the clinical trial was registered (ClinicalTrials.gov ID: NCT02852616); the study protocol is also available from the authors. This study was conducted in Seoul, the capital of South Korea, between July 2018 and April 2019. All participants were recruited through an alternative school for youth from North Korea and children of North Korean refugees who were born in China. A total of of 81 students were screened before admission into the study. Inclusion criterion was a diagnosis of PTSD according to criteria in the fifth edition of the *Diagnostic and Statistical Manual of Mental Disorders* (*DSM‐5*). Exclusion criteria were severe psychotic disorder, neurological disorder, acute suicidal behavior, or intellectual disabilities. All participants, and parents for all minors, provided written informed consent.

The NET condition comprised five to 10 individual sessions (*M* = 8 sessions), which were 90–120 min each. The NET intervention was performed in accordance with the manual, as outlined by Schauer et al. (2011) and its Korean translation (Choi & Park, 2014). The first author, a clinical psychologist who was trained in NET and previously delivered treatment to traumatized individuals, carried out the treatment in Korean. Supervision included documentation of cases and communications by e‐mail or telephone. In the TAU condition, participants continued to receive their usual care, which included supportive therapy or art therapy depending on the needs determined by the institution. The TAU condition comprised 10–15 individual sessions of 40–60 min duration each as well as one session that covered sleep education, during which participants were educated in a 1‐hr, psychiatrist‐led group session about the nature, prevalence, prevention, and intervention of sleep disorders. Pretest assessments (t0) were conducted in Korean by a clinical psychologist as well as a counseling professor who worked with traumatized individuals. To test the immediate effects of NET, a posttest assessment was conducted 2 weeks after treatment completion (t1) for the NET group. For both groups, follow‐up time points were 3 (t2) and 6 months (t3) after the end of treatment. To reduce potential sources of assessment bias, follow‐up assessments were conducted by a trained researcher who had not participated in the therapy portion of the study. If participants in the TAU group still fulfilled the criteria for PTSD at the last follow‐up examination, they were immediately offered NET.

### Measures

#### PTSD diagnosis and symptoms

Diagnosis and severity of PTSD were assessed using the UCLA PTSD Index for *DSM‐5* (UPID; Elhai et al., 2013; Steinberg et al., 2013), which is the revision of the UCLA Child/Adolescent PTSD Reaction Index for *DSM‐IV*. The UPID includes 27 items designed to measure PTSD symptoms as well as four items that assess the PTSD dissociative subtype. Respondents rate each item on a scale of 0 (*none*) to 4 (*most of the time*), based on past‐month symptom frequency. Severity of PTSD was calculated by summing the scores for all items related to PTSD symptoms, with a cutoff score of 38 (range: 0–80). In the present sample, Cronbach's alpha was .81.

#### Insomnia

Insomnia symptoms were evaluated using the seven‐item Insomnia Severity Index (ISI; Bastien, Vallières, & Morin, 2001), which assesses sleep difficulties over the last 2 weeks, using a scale of 0 (*none*) to 4 (*very severe*). The total sum score is defined as the severity of insomnia and interpreted as follows: none (0–7), subthreshold (8–14), moderately severe (15–21), and severe insomnia (22–28). In the present sample, the Cronbach's alpha value was .79.

#### Sleep quality

Sleep quality was assessed using the 19‐item Pittsburgh Sleep Quality Index (PSQI; Buysse, Reynolds, Monk, Berman, & Kupfer, 1989), which contains items related to past‐month sleep quality and disturbances. Respondents are asked to rate items on a scale of 0 (*not during the past month*) to 3 (*three or more times a week*). In the present sample, Cronbach's alpha was .76.

#### Depression

Depression symptoms were assessed using nine‐item Patient Health Questionnaire (PHQ‐9; Kroenke, Spitzer, & Williams, 2001), which is designed to measure the severity of depression over the last 2 weeks. Respondents are asked to rate items on a scale of 0 (*not at all*) to 3 (*nearly every day*). Scores are summed to indicate depression severity, with higher scores representing a higher level of severity. In the present sample, Cronbach's alpha was .80.

#### Internalizing and externalizing symptoms

Internalizing and externalizing symptoms were measured using the 25‐item Strengths and Difficulties Questionnaire (SDQ; Goodman, 1997), which consists of five subscales: Emotional Symptoms, Peer Problems, Conduct Problems, Hyperactivity, and Prosocial Behavior. Each subscale includes five items, which respondents are asked to rate on a scale of 0 (*not true*) to 2 (*certainly true*). In the present study, symptom severity was generated by summing the scores from all the scales except the Prosocial subscale (range: 0–40). The Cronbach's alpha value in the present sample was .77.

### Data Analysis

Data analyses were performed using SPSS (Version 25.0). Group differences for sociodemographic and clinical variables at baseline were examined using Fischer's exact test and *t* tests. For outcome measures, linear mixed‐effects models were used to analyze changes from t0 to t3. To evaluate the development of clinical symptoms between pretreatment and follow‐ups, we calculated a series of repeated‐measurement analyses of variance (ANOVAs) for the PTSD severity score and the scores for the different symptoms, with treatment condition (NET, TAU) as the between‐group factor and time point (t0, t2, t3) as the repeated‐measures factor. Changes from t0 to t2 and t3 within the NET group were calculated by linear mixed models as well as paired *t* tests for post hoc comparisons. A Bonferroni correction was applied to control the family‐wise error rate and compute the adjusted *p* values. Mauchly's tests were performed for testing the sphericity assumption. For cases in which the assumption was violated, the Greenhouse‐Geisser correction was used. In the NET group, one participant completed the 2‐week and 3‐month follow‐up interviews but did not participate in the 6‐month follow‐up. We included this participant in the analysis by using the “last observation carried forward” method. Hedge's *g* was used to calculate effect sizes for both conditions regarding overall symptoms, and eta squared was calculated as an effect size for the omnibus ANOVAs.

## Results

### Comparison of NET and TAU Over Time

**Figure 1:**
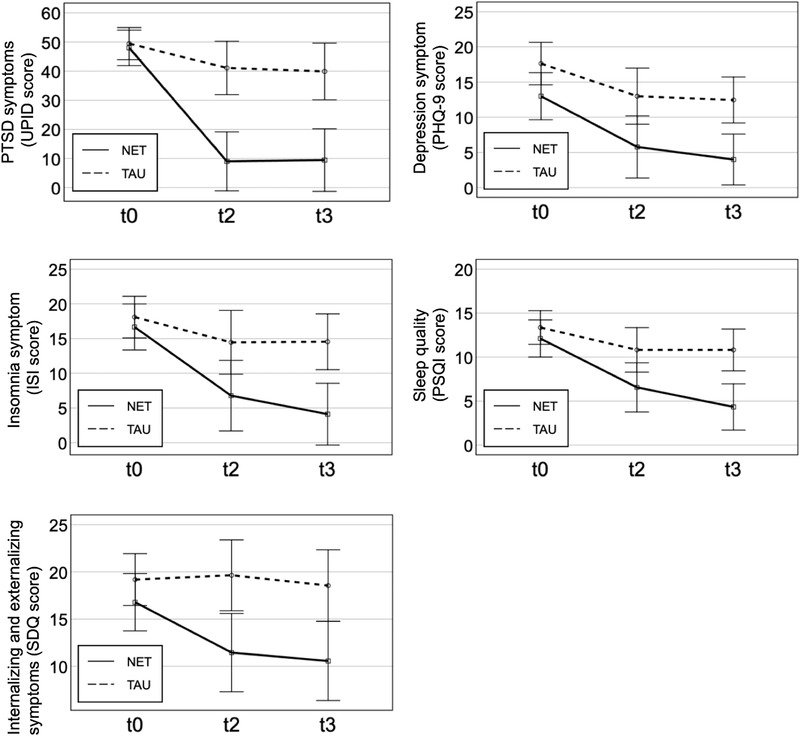
Changes in symptom severity before treatment and at follow‐ups in the narrative exposure therapy (NET; *n* = 9) and treatment‐as‐usual (TAU; *n* = 11) groups. Bars represent 95% confidence intervals. PTSD = posttraumatic stress disorder; t0 = pretreatment; t2 = 3‐month follow‐up; t3: 6‐month follow‐up; UPID = UCLA PTSD Index; PHQ = Patient Health Questionnaire; ISI = Insomnia Severity Inventory; PSQI = Pittsburgh Sleep Quality Index = SDQ: Strengths and Difficulties Questionnaire.

There was a significant group difference in scores for all clinical characteristics (see Table 2 and Figure 1). For PTSD symptom severity, we found a significant main effect of time, *F*(1.48, 36) = 38.28, *p* < .001, η_p_^2^ = .68; treatment, *F*(1, 18) = 20.89, *p* < .001, η_p_^2^ = .54; and the Group x Time interaction, *F*(1.48, 36) = 14.97, *p* < .001, η_p_^2^ = .45. Symptoms of PTSD were significantly reduced in the NET group from t0 to t3, Hedges’ *g* = 3.57, *p* < .001; but not in the TAU group, Hedges’ *g* = 0.70, *p* = .382. Six months after treatment, five of the 11 participants in the TAU group still met the criteria for a PTSD diagnosis, whereas all participants of the NET group had lost their diagnostic status, Fischer's exact test *p* = .038. Regarding the UPID cutoff score for PTSD, six participants in the TAU group (54.5%) scored at or above the cutoff of 38 as compared to only one participant in the NET group (11.1%). For insomnia, we found significant main effects for Group x Time, *F*(1.36, 36) = 7.13, *p* = .008, η_p_^2^ = .28; time, *F*(1.36, 36) = 25.04, *p* < .001, η_p_^2^ = .58; and treatment, *F*(1, 18) = 7.35, *p* = .014, η_p_^2^ = .29. Participants in the NET group showed a substantial decline in insomnia symptoms from t0 to t3, Hedges’ *g* = 2.14, *p* = .001; but those in the TAU group did not, Hedges’ *g* = 0.66, *p* = .211. Regarding sleep quality, a substantial improvement was found in among participants in the NET group from t0 to t3, Hedges’ *g* = 2.16, *p* < .001, but not among those in the TAU group, Group x Time: *F*(2, 36) = 4.02, *p* = .027, η_p_^2^ = .18; time: *F*(2, 36) = 17.22, *p* < .001, η_p_^2^ = .49; and treatment: *F*(1, 18) = 10.71, *p* = .004, η_p_^2^ = .37.

**Table 2:** Changes in Clinical Characteristics in the Narrative Exposure Therapy (NET) and Treatment as Usual (TAU) Groups

	Pretest		2‐week follow‐up		3‐month follow‐up		6‐month follow‐up		Statistical test	
Group	*M*	*SD*	*M*	*SD*	*M*	*SD*	*M*	*SD*	Group × Time	Main effect of time for NET
PTSD symptoms										
NET	48.0	6.44	11.33	10.49	9.0	12.53	9.44	13.87	14.97***	76.41***
TAU	49.45	10.13	–		41.09	15.82	39.91	16.45		
Insomnia symptoms										
NET	16.67	4.72	10.56	4.72	6.78	7.34	4.11	6.83	7.13**	20.66***
TAU	18.09	4.74	–		14.45	7.17	14.55	5.94		
Sleep quality										
NET	12.11	2.89	6.56	3.13	6.56	4.61	4.33	4.21	4.02*	10.94**
TAU	13.36	3.11	–		10.81	3.43	10.82	3.34		
Depression symptoms										
NET	13.0	6.0	4.11	4.08	5.78	7.1	4.0	6.02	1.12	10.21**
TAU	17.64	3.5	–		13.0	5.59	12.45	4.34		
Internalizing and externalizing symptoms										
NET	16.78	5.24	9.56	4.88	11.44	7.81	10.56	7.14	4.89*	8.76***
TAU	19.18	3.46	–		19.64	3.78	18.55	4.84		

*Note*. PTSD = posttraumatic stress disorder.

* *p* < .05, **p < .01, ***p < .001.

A repeated‐measure ANOVA for depression revealed no significant interaction of group and time, *F*(1.38, 36) = 1.12, *p* = .322, η_p_^2^ = .06, but there was a significant main effect of both time, *F*(1.38, 36) = 17.07, *p* < .001, η_p_^2^ = .49; and treatment, *F*(1, 18) = 12.25, *p* = .003, η_p_^2^ = .41, whereby a significant reduction from t0 to t3 was found for both, Hedges’ *g* = 1.50, *p* = .011, for the NET group; Hedges’ *g* = 1.31, *p* = .020 for the TAU group. At the symptom level, however, there were significant results at various follow‐up time points, depending on type of treatment. Compared to participants in the TAU group, those who received NET reported significantly lower depression scores at t2 (*M* = 13, *SD* = 5.6 vs. *M* = 5.8, *SD* = 7.1, respectively), *p* = .020, and t3 (*M* = 12.5, *SD* = 4.3 vs. *M* = 4, *SD* = 6, respectively), *p* = .002. For internalizing and externalizing symptoms, we found a significant Group x Time interaction, *F*(2, 36) = 4.89, *p* = .013, η_p_^2^ = .21. There was a significant reduction in symptoms from t0 to t3 among participants in the NET group, Hedges’ *g* = 0.99, *p* = .042, but not in the TAU group, Hedges’ *g* = 0.15, *p* = 1.00. The results remained statistically significant after correcting for multiple testing. Between‐condition effects were medium in magnitude for depression and large for all other symptoms.

### Changes in the NET Group Over Time

There was a significant time effect for all symptoms among participants in the NET group (see Table 2) for PTSD, *F*(3, 24) = 76.41, *p* < .001, η_p_^2^ = . 91; insomnia, *F*(3, 24) = 20.66, *p* < .001, η_p_^2^ = .72; depression, *F*(1.69, 24) = 10.21, *p* = .003, η_p_^2^ = .56; sleep quality, *F*(2.04, 24) = 10.94, *p* = .001, η_p_^2^ = .58; and internalizing and externalizing symptoms, *F*(3, 24) = 8.76, *p* < .001, η_p_^2^ = .52. Significant reductions, with large effects, were found between t0 and t1 for PTSD, *p* < .001; insomnia, *p* = .042; sleep quality, *p* = .025; and depression, *p* = .012. Similar results were observed between t0 and t3 for the same variables, with *p* values of < .001, .001, .010, and .023, respectively. For internalizing and externalizing symptoms, changes between t0 and t1 and between t0 and t2 were significant, *p* < .001 and *p* = .018, respectively. Overall, NET elicited a substantial reduction that was observed shortly after treatment completion, and its effect was stable for at least 6 months posttreatment (see Figure 1).

## Discussion

The present study investigated the effectiveness of NET in the treatment of traumatized North Korean refugee youth. Consistent with the reported effects of NET on traumatized populations (Neuner, Schauer, & Elbert, 2018), we found a clinically significant reduction in PTSD, depression, and internalizing and externalizing symptoms among participants in the NET group. The change in the diagnostic status for PTSD was more notable in participants who were given NET than those who engaged in TAU. Regarding depression, it is important to note that although a symptom reduction was observed in both the NET and TAU conditions, the reduction found in the NET group was significantly larger and clinically important, and participants in the TAU group reported clinical levels of remaining PTSD and depression symptoms after treatment completion. Narrative exposure therapy is an evidence‐based, trauma‐focused treatment that contextualizes traumatic memories in time and space, reducing the perceived threat and stress by the reconstruction of a coherent life narrative (Schauer et al., 2011). Given the strong treatment effect found for NET on both PTSD and depression symptoms in the present study, we propose that proper processing of traumatic memories resulted in the reduction of not only PTSD but also depression for participants in the NET group. The present findings suggest that NET, with its trauma‐focused component, appears to be more effective than TAU in the treatment of depression in North Korean refugee youth with PTSD.

Additionally, NET produced a significant improvement in insomnia symptoms and sleep quality, whereas TAU did not. Our results concur with reported findings that have shown CBT to be more effective than non–trauma‐focused therapy for treating sleep problems in individuals with PTSD (Brownlow et al., 2016; Woodward et al., 2017) as well as findings that have demonstrated changes in some sleep parameters after NET (Weinhold et al., 2017). The study by Weinhold et al. (2017), however, found no effect of NET on the subjective data of the PSQI, which we used to assess sleep quality in the present study. One possible explanation for this inconsistent result may be the different sample populations (i.e., medicated clients with severe BPD vs. unmedicated refugee youth with PTSD. Considering previous evidence showing that PTSD may provide a link, via depression, between trauma exposure and insomnia in North Korean refugee youth (Park et al., 2019), the therapeutic improvement in sleep problems we found in the NET group seems to be associated with the reduced PTSD and depression symptoms achieved through trauma‐focused intervention. This assumption is supported by our findings of residual sleep problems in the TAU group despite improvement in depression symptoms and a session on sleep education as well as by congruent results observed in previous studies, which have indicated an association between sleep disturbance and PTSD symptom severity (Belleville, Guay, & Marchand, 2011; Brownlow et al., 2016). Our findings suggest that an improvement in sleep may indicate successful treatment outcome concerning PTSD. The fact that the NET group showed no remaining clinically relevant sleep problems after treatment is incongruent with some previous evidence that has shown residual sleep difficulties to be observed in treatment responders after successful treatment of PTSD (Belleville et al., 2011; Brownlow et al., 2016; Gutner et al., 2013). In their review of the treatment of sleep disturbances in PTSD, Schoenfeld, DeViva, and Manber (2012) pointed out that additional interventions may be required to address posttraumatic sleep problems even after successful treatment. We suggest further studies to replicate and extend the present findings to understand the ways in which the treatment component and mechanisms of NET affect sleep.

The present study had some limitations worth mention. Most notably, the sample size was small, and there was a lack of random group assignment. Due to the limited sample size, we were not able to control the preexisting group difference in depression nor could we assess whether depression was a mediator or moderator of the treatment effects.

In conclusion, the results of our study suggest that North Korean refugee youth with PTSD can be treated effectively through trauma‐focused intervention in the short term. Among the present sample, NET was more effective than TAU for the treatment of posttraumatic symptoms in North Korean refugee youth, and the NET intervention demonstrated lasting benefits. Additionally, this was, to date, the first study of which we are aware to demonstrate the effect of NET on changes in sleep patterns in traumatized refugees. The results of the present study suggest that NET appears to be a treatment option for traumatized North Korean refugee youth and may also be effective for the treatment of sleep problems in relation to traumatic experiences.
